# Effect of *Periplaneta americana* Residue Feed on Immunity, Antioxidant Capacity, and Transcriptome in Chickens: A Study on Sanhuang Chickens

**DOI:** 10.3390/ani15010094

**Published:** 2025-01-03

**Authors:** Yongfei Zhao, Tianzhang Zhao, Shunyi Zi, Guoyu Ou, Huiying Li

**Affiliations:** 1 College of Agriculture and Biological Sciences, Dali University, Dali 671000, China; 18313039035@163.com (Y.Z.); zhaotianzhang@dali.edu.cn (T.Z.); 15087837679@163.com (S.Z.); 15125471774@163.com (G.O.); 2 Yunnan Provincial Key Laboratory of Entomological Biopharmaceutical R&D, College of Pharmacy, Dali University, Dali 671000, China; 3 National-Local Joint Engineering Research Center of Entomoceutics, Dali University, Dali 671000, China

**Keywords:** *Periplaneta americana*, immune function, inflammation, antioxidant capacity, transcriptome

## Abstract

This study investigated the effects of *Periplaneta americana* residue (PAR) on the immune function, antioxidant capacity, and transcriptome of Sanhuang chickens. Six hundred 30-day-old Sanhuang chickens were divided into six groups with diets of varying PAR replacement of soybean meal from 0 to 100% in increments of 20%. Samples were taken on day 100. The results indicated that PAR did not significantly affect growth performance (*p* > 0.05). PAR significantly elevated the levels of IL-1β, IL-2, TNF-α, IgA, IgG, and IgM in both the serum and spleen (*p* < 0.05). Moreover, it markedly increased the GSH-Px, T-AOC, SOD, and CAT levels in the serum and liver (*p* < 0.05) and reached optimal levels of immune factors and antioxidant indicators at diet 2. Transcriptomic analysis revealed that substituting PAR for soybean meal downregulated genes associated with immune diseases and infectious disease pathways. Substituting soybean meal with PAR enhanced the resistance of Sanhuang chickens to pathogenic factors and oxidative stress, with no impact on growth performance. The optimal improvement was observed with diet 2.

## Simple Summary

The polypeptides extracted from *Periplaneta americana* have been found to possess immune-regulating, anti-oxidative stress, and antimicrobial effects. *Periplaneta americana* residue (PAR) is a by-product obtained after the pharmaceutical extraction process of *Periplaneta americana*. In this study, different feeds with varying contents of PAR were used to feed Sanhuang chickens and the immune function (serum and spleen) and antioxidant capacity (serum and liver) of the Sanhuang chickens were tested. It was found that an appropriate amount of PAR could improve the immune and antioxidant functions of Sanhuang chickens. Through transcriptomic sequencing, it was discovered that PAR regulates the gene expression in the spleen of Sanhuang chickens. Enrichment analysis revealed that these differentially expressed genes were significantly enriched in terms and pathways related to pathogenic factor invasion. These results provide scientific evidence for the application of PAR as a supplement in Sanhuang chickens.

## 1. Introduction

Researchers have proposed using insect protein as a substitute for fishmeal and soybean meal to address protein resource shortages [1]. *Periplaneta americana* (PA), which has a high protein content, has been studied as a source of protein in animal feed [2]. PA is processed into feed as a protein source and used to feed chickens [3], quail [4], fishes [5], and sheep [6]. Replacing fish meal with PA in the basic diet can significantly improve the immune function, antioxidant capacity, and intestinal antimicrobial ability of male broilers and has the function of promoting growth and enhancing disease resistance [7]. Long et al. [5] also achieved the same results by using PA as a substitute for fish meal in fish feed. Additionally, PA powder as an additive can reduce the feed conversion ratio (FCR) of broilers and improve the immune organ index and antioxidant capacity of broilers [8]. In summary, PA represents a promising alternative protein source for animal feed, demonstrating significant benefits in terms of growth performance, immune function, and antioxidant capacity across various species. However, due to the unstable yield of wild PA and the significantly higher cost of artificially reared American cockroaches compared to soybean meal, the application of PA in insect protein is limited [9].

In recent years, various peptides derived from PA, including periplanetasin-2 [10], periplanetasin-4 [11], periplanetasin-5 [12], Pa-THY1 [13], and sNPF [14], have garnered attention for their medicinal properties. PA is specifically bred for the extraction of pharmacologically active ingredients, which are used in the manufacture of medicines such as Kangfu Xin and Xinmai Long [15]. Interestingly, the protein content of *P. americana* residue (PAR) remains largely unchanged even after the drug extraction procedures. The analysis of the PAR’s basic nutritional components shows that it contains 55.91% crude protein and 20.48% crude fat (dry matter). In addition, PAR has a significant advantage over other insect proteins. PAR is a byproduct of the pharmaceutical manufacturing process, so we can obtain it at a significantly lower price than other insects. Furthermore, owing to the demand for medications like Kangfu Xin and Xinmai Long, PAR has a relatively stable production output. However, due to the lack of scientific evidence, PAR has not been fully utilized. Consequently, PAR is often disposed of through composting, incineration, and by providing it to farmers near the pharmaceutical plants for animal feed. These methods, due to technical and channel limitations, currently do not generate revenue for the pharmaceutical plants and may result in certain financial expenditures and resource wastage. Long et al. [16] reported that the excessive inclusion of PAR in feed can negatively impact the feed conversion ratio; this indicates that using PAR directly for animal feed may result in economic losses for farmers. Therefore, it is necessary to seek scientific and efficient methods for the utilization of PAR.

Poultry in outdoor environments instinctively capture and consume insects, indicating that their digestive systems are adapted to insects as part of their diet. Research by Kovitvadhi et al. [17] revealed that the digestive fluids of chickens exhibit efficient digestion of PA, suggesting that PAR could serve as a promising protein source in poultry feed.

Sanhuang chicken (three yellow chicken) is a popular native breed in South China and widely eaten meat due to its fatty skin, delicious flavor, and soft and tender flesh [18]. The preliminary research of this laboratory found that 20% and 40% of PAR in feed have positive effects on the growth performance, digestive enzyme activity, intestinal morphology, intestinal flora, and nutrient digestion and absorption of the Sanhuang chicken [19]. Throughout their growth stages, chickens inevitably encounter various immune challenges stemming from viruses, pathogens, and parasites [20,21,22]. While previous studies have demonstrated the immune-boosting and antioxidant-enhancing effects of PAR in fish [16], the specific impact of PAR on the immune and antioxidant functions of chickens remains unexplored.

Recent advancements in transcriptomic sequencing technologies have provided valuable insights into the molecular mechanisms underlying the immune and physiological responses of animals to dietary interventions and environmental stimuli [23,24]. Transcriptomic analysis allows for the comprehensive examination of gene expression patterns, which can reveal the intricate networks regulating immune function, antioxidant defense, and growth performance in poultry [25]. Previous studies have shown that different dietary compositions can lead to varying expressions of genes related to immune responses [26]. The spleen is one of the most important immune organs in chickens. Therefore, we aim to use transcriptomic sequencing to understand the regulation of PAR on genes associated with immune responses.

Given previous findings, we hypothesize that PAR may play a significant role in modulating immune and antioxidant responses in chickens. To test this hypothesis, this study conducted the detection of immune factors in serum and the spleen, as well as antioxidant indices in serum and the liver. In addition, transcriptomic sequencing was employed to sequence the spleens of chickens fed with different PAR-content feeds, systematically investigating the effects of PAR on the immune and antioxidant functions of Sanhuang chickens.

## 2. Materials and Methods

This study was carried out in accordance with the recommendations of the Care and Use of Laboratory Animals in China, Animal Ethical and Welfare Committee of China Experimental Animal Society. The protocol was approved by the Animal Ethical and Welfare Committee of Dali University (No. 2023-PZ-275).

### 2.1. PAR Treatment

PAR, the residue of PA after the effective medicinal ingredients are extracted by ethanol, was obtained from Yunnan Tengyao Pharmaceutical Co., Ltd., which is located in Tengchong, Yunnan Province, China. The PAR was dried at 90 °C and ground to pass through an 80 mesh screen, ensuring that the particle size was less than 0.18 mm for later use. In addition, we examined the antimicrobial peptides and total polysaccharide content of the PAR.

The detection of active substances in the PAR was conducted according to the following procedure. PAR samples (0.2 g) were homogenized in 1.8 mL of phosphate-buffered saline and centrifuged at 3000× *g* at 4 °C for 10 min. Then, the supernatant was collected for analysis. PAR periplanetasin-2, periplanetasin-4, defensins, periplanetasin-5, Pa-THY1, sNPF, attacins, lysosomes, and antimicrobial peptide levels were detected using enzyme-linked immunosorbent assay (ELISA) kits (MLBIO Co., Shanghai, China). The ELISA utilizes the specific binding of antibodies to antigens to accurately identify and quantify target peptides, making it suitable for the detection of target molecules with low abundance in samples [27]. The main steps for an ELISA are as follows: (1) standard, sample diluent; (2) add standard, sample diluent, incubate for 30 min at 37 °C; (3) wash 5 times, add HRP-conjugated reagent, incubate for 30 min at 37 °C; (4) wash 5 times, add chromogen solutions A and B, incubate for 10 min at 37 °C; (5) add stop solution; (6) read absorbance at 450 nm within 15 min; and (7) calculate [28]. The same kit and method will be used for the subsequent detection of immune factors and antioxidant indicators, and will not be re-stated.

The total polysaccharides were extracted using the water extraction and alcohol precipitation method [29] and their levels were determined using total polysaccharide assay kit (MLBIO Co., Shanghai, China). The main steps are as follows: (1) standard, sample diluent; (2) add standard, sample diluent, incubate for 20 min at 90 °C; (3) add HRP-conjugated reagent, incubate for 30 min at 37 °C; (4) read absorbance at 490 nm within 15 min; and (5) calculate. All determination procedures were performed strictly according to the manufacturer’s instructions. The inter- and intra-assay coefficients of variation (CVs) were less than 10%.

### 2.2. Dietary Treatments and Management of Experimental Animals

A total of 600 30-day-old Sanhuang chickens were randomly assigned to six dietary treatments, with ten replicates in each treatment and ten chickens in each replicate (five male and five female). Each replicate of chickens was raised in a cage (length × width × height, 128 cm × 60 cm × 50 cm). The chickens were fitted with numbered tags and their weights were recorded before the experiment, and there were no significant differences in initial body weight among the treatments (*p* > 0.05).

By analyzing the nutritional components of the PAR, it was found that it contained 55.91% crude protein and 20.48% crude fat. Based on previous research results, there is no significant difference in the growth performance of Sanhuang chickens between the groups fed with PAR replacing soybean meal (at 20%, 40%, 60%, 80%, and 100%) and the control group [19]. However, the diet required for individuals to achieve optimal immune capacity and antioxidant status is often not the same as the diet required for optimal growth performance. To initially determine the PAR level most beneficial for the immune function and antioxidant status of Sanhuang chickens, further research is needed. Experimental diets with equal nitrogen and lipid contents were prepared using 0%, 4.20%, 8.28%, 12.32%, 16.4%, and 20.58% PAR to replace 0%, 20%, 40%, 60%, 80%, and 100% soybean meal, respectively, in the basal diet, with appropriate adjustments to the other ingredients. The ingredients in the diets and the nutritional composition are shown in Table 1.

Throughout the entire experiment, the chickens in each group had ad libitum access to feed and water and the experimental diets were provided in powdered form. During the first week, 24 h of light was provided, after which the light was reduced by 2 h per week and maintained at 18 h until the end of the experiment. During the first week, the house temperature was maintained at 32 °C and then reduced by 2 °C per week until it reached 26 °C. No medication was administered to the chickens during the experiment and the weights and identification numbers of any chickens that died during the experiment were recorded.

### 2.3. Measurement of Growth Performance

On day 100, all chickens were weighed and their final weights and feed consumption were recorded. Subsequently, the FCR was computed using the following formula: FCR = average daily feed intake (ADFI) (g)/average daily gain (ADG) (g).

### 2.4. Sample Collection and Randomization

On day 72, one medium-weight chicken was selected from each replicate of each treatment for sample collection, totaling 60 chickens. After fasting for 6 h, the chickens were weighed and slaughtered. Blood samples were collected in heparinized tubes from the slaughtered hens and centrifuged at 3000× *g* for 4 °C to obtain serum, which was then stored at −20 °C for subsequent use. The spleens and livers of the chickens were manually removed and stored at −80 °C for later analysis. It is worth mentioning that we collected two spleen samples from each chicken, one for transcriptome analysis and the other for biochemical analysis. All samples were subjected to a simple randomization process by a member not involved in the testing before the analysis. This person renumbered all the sample identifiers and was responsible for compiling and summarizing the experimental results after the testing was completed.

### 2.5. Assay of Immune Parameters in the Serum and Spleen

Referring to the method of Liu et al. [28], ELISA kits were used to detect immune factors. Serum samples were processed by a vortex oscillator for 30 s and centrifuged at 2000× *g* at 4 °C for 10 min. Spleen samples (0.2 g) were homogenized in 1.8 mL of phosphate-buffered saline and centrifuged at 3000× *g* at 4 °C for 10 min. Then, the supernatant was collected for analysis. Serum and spleen interleukin 1 beta (IL-1β), interleukin 2 (IL-2), tumor necrosis factor-alpha (TNF-α), immunoglobulin A (IgA), immunoglobulin M (IgM), and immunoglobulin G (IgG) levels were detected using ELISA kits (MLBIO Co., Shanghai, China). All determination procedures were performed strictly according to the manufacturer’s instructions. The inter- and intra-assay coefficients of variation (CVs) were less than 10%.

### 2.6. Assessment of Oxidative Status in the Serum and Liver

Referring to the method of Onorato et al. [31], ELISA kits were used to detect antioxidant indicators. Serum samples were processed by a vortex oscillator for 30 s and centrifuged at 2000× *g* at 4 °C for 10 min. Liver samples (0.2 g) were homogenized in 1.8 mL of phosphate-buffered saline and centrifuged at 3000× *g* at 4 °C for 10 min. Then, the supernatant was collected for detection by ELISA. The oxidative status of serum and hepatic homogenates was evaluated by determining malondialdehyde (MDA) levels, total antioxidant capacity (T-AOC), catalase (CAT) capacity, total superoxide dismutase (SOD) activity, and glutathione peroxidase (GSH-Px) capacity. All determination procedures were performed strictly according to the manufacturer’s instructions. The inter- and intra-assay coefficients of variation (CVs) were less than 10%.

### 2.7. Spleen Transcriptome Assay

Due to the chicken cages being divided into upper, middle, and lower layers, and there being certain differences in the environments of these layers, to reduce the impact of environmental factors, we ensured that chickens from different replicates were as evenly distributed as possible across the upper, middle, and lower layers during rearing. The spleen transcriptome samples for each group were respectively collected from the upper, middle, and lower layers. Total RNA from the spleen tissues (3 replicates per treatment) was extracted with TRIzol^®^ Reagent according to the manufacturer’s instructions. Then, RNA quality was determined by a 5300 Bioanalyzer (Agilent, Santa Clara, CA, USA). RNA purification, reverse transcription, library construction, and sequencing were performed at Shanghai Majorbio Biopharm Biotechnology Co., Ltd. (Shanghai, China) according to the manufacturer’s instructions (Illumina, San Diego, CA, USA). The raw paired-end reads were trimmed and quality controlled by fastp [32] with default parameters. Then, the clean reads were separately aligned to the reference genome (*Gallus gallus*) in orientation mode using HISAT2 2.2.1 [33] software. RNA-Seq by Expectation Maximization was used to quantify gene abundances [34]. To identify DEGs (differential expression genes) between two different samples, the expression level of each transcript was calculated according to the transcripts per million reads (TPM) method. Differential expression analysis was performed using the DESeq2 and DEGs with |log2FC| ≥ 1 and FDR ≤ 0.05 (DESeq2) were considered to be significantly differentially expressed genes [35].

In addition, functional enrichment analysis, including Gene Ontology (GO) and the Kyoto Encyclopedia of Genes and Genomes (KEGG), was performed to identify which DEGs were significantly enriched in GO terms and metabolic pathways at Bonferroni-corrected *p* values ≤ 0.05 compared with the whole-transcriptome background. The Bonferroni correction calculates adjusted *p*-values by dividing the desired family-wise error rate (α) by the number of comparisons (k) and a result is considered significant if its *p*-value is less than or equal to α/k. These DEGs were then mapped to the *Gallus gallus* reference genome employing HISAT2, with functional annotations conducted using databases such as KEGG. GO functional enrichment and KEGG pathway analyses were carried out by Goatools [36] and KOBAS [37], respectively.

Gene set enrichment analysis (GSEA) was used to examine the overall gene expression of the KEGG pathways enriched in the DEGs between the experimental group and control group, and the calculation of the enrichment score was based on the method proposed by Subramanian et al. [38].

The expression data of spleen tissues in diets 1, 2, 3, 4, 5, and 6 were subjected to weighted gene coexpression network construction using the weighted gene coexpression network analysis (WGCNA) package and following the pipeline [39]. To identify modules that were significantly associated with the levels of IL-1β, IL-2, TNF-α, IgA, IgG, and IgM in the spleen, Pearson correlations (*p* < 0.05 and |r| > 0.4) were carried out. The top 200 hub genes in each module were chosen by the Connectivity function.

GO analysis, KEGG analysis, GSEA, and WGCNA were all conducted using the Majorbio Cloud Platform: https://login.majorbio.com (accessed on 20 December 2024). The raw data were uploaded to the NCBI SRA database (sequence number: PRJNA1080808).

### 2.8. Statistical Analysis

Statistical analyses were conducted using one-way analysis of variance (ANOVA) and linear and quadratic regression with Duncan’s multiple comparisons. *p* < 0.05 was regarded as the level of statistical significance. In addition, an effect size analysis was conducted on immune factors and antioxidant indicators to investigate the impact of PAR on their levels. These statistical analyses were performed using IBM SPSS Statistics 27.

## 3. Results

### 3.1. The Peptide Content in PAR

According to the test results (Table 2), the PAR contained 13.93% total polysaccharides (dry matter). The PAR contained 13.43 µg/L of lysosome, 85.22 ng/L of sNPF, 1338.53 ng/L of defensins, 135.93 pg/mL of periplanetasin-2, 87.21 pg/mL of periplanetasin-4, 52.97 pg/mL of periplanetasin-5, 34.67 pg/mL of attacins, and 201.27 pg/mL of antimicrobial peptides (containing 44.78% moisture).

### 3.2. Growth Performance

As shown in Table 3, the PAR had no significant effect on the ADG, ADFI, and FCR (*p* > 0.05). An increase in the level of PAR linearly (*p* = 0.009) and quadratically (*p* = 0.012) affected the ADG, which was significantly greater in diet 3 than in diet 6 (*p* < 0.05). An increase in the level of PAR linearly (*p* = 0.010) and quadratically (*p* = 0.017) affected the FCR, which was significantly greater in diet 6 than in diet 2 and diet 3 (*p* < 0.05). It is worth mentioning that there was no significant difference in growth performance between diet 6 and diet 1.

### 3.3. Immune Parameters in the Serum and Spleen

As shown in Table 4, PAR intake showed a quadratic increase in spleen IgG concentration (*p* < 0.001) and a tendency to increase spleen Il-2 and IgA concentration quadratically (*p* = 0.062 and *p* = 0.089, respectively). The concentrations of IL-1β, IL-2, TNF-α, IgA, IgG, and IgM in the spleen and serum of the experimental groups were greater than those in the control group, with diet 2 showing the highest concentration. Additionally, there was a difference (*p* < 0.05) in these immune indicators between diet 1 and diet 2. The analysis of effect size shows that the Eta squared values are all greater than 0.70, indicating a strong influence of the PAR on the levels of immune factors in the spleen and serum, explaining more than 50% of the variability.

### 3.4. Oxidative Capacity in Serum and Liver

As shown in Table 5, increasing the PAR in the diet resulted in a quadratic increase in liver GSH-Px (*p* = 0.021) concentration and serum SOD activity (*p* = 0.018), as well as a quadratic decrease in serum MDA content (*p* = 0.023). There was a tendency for the serum CAT concentration to increase quadratically (*p* = 0.067). The activities of GSH-Px, T-AOC, SOD, and CAT in the liver and serum of the experimental groups were greater than those in the control group, with diet 2 showing the greatest increase. The content of MDA in the liver and serum of the experimental groups was lower than that in the control group, with diet 2 showing the lowest MDA content. Additionally, there was a difference (*p* < 0.05) in the levels of these antioxidants between diet 1 and diet 2. The Eta squared values for the liver and serum immune indices were 0.590, 0.819, 0.904, 0.376, and 0.726 and 0.681, 0.867, 0.854, 0.809, and 0.839, respectively, all indicating strong effects and explaining a substantial portion of the variability.

### 3.5. Differentially Expressed Genes

**Figure 1:**
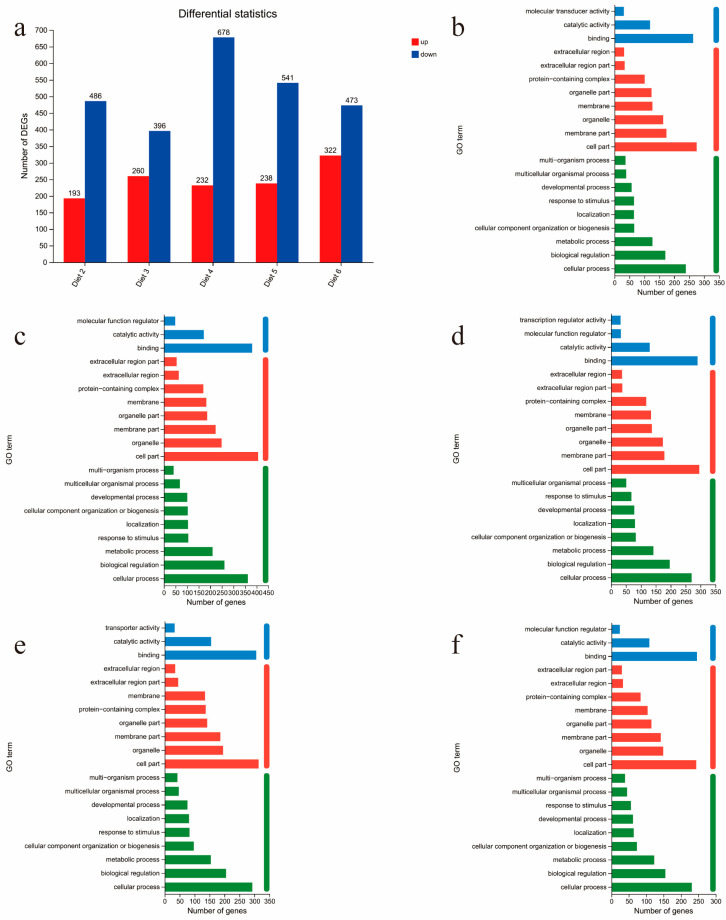
DEG and GO annotation analysis. (**a**) DEGs of the experimental group and control group. (**b**–**f**) The top 20 entries annotated in the GO database for DEGs in the experimental and control groups, (**b**–**f**) are Diet 2, Diet 3, Diet 4, Diet 5, and Diet 6, respectively.

To investigate the effects of replacing expanded soybean meal in feed with different doses of PAR on Sanhuang chickens, we conducted RNA-seq analysis to examine the gene expression profile. Compared to diet 1, a total of the results of 679 (193 upregulated; 486 downregulated), 656 (260 upregulated; 396 downregulated), 910 (232 upregulated; 678 downregulated), 779 (238 upregulated; 541 downregulated), and 795 (322 upregulated; 473 downregulated) DEGs were identified in diet 2, 3, 4, 5 and 6, respectively (Figure 1a). Simultaneously, 145 (29 upregulated; 116 downregulated) DEGs were shared among five groups. Including *HSPB9* (heat shock proteins), this gene was downregulated in all five experimental groups.

### 3.6. GO Annotation and GO Enrichment Analysis of DEG

These DEGs were classified by GO annotation into three main categories: biological process, cellular component, and molecular function. In the molecular function ontology, binding and catalytic activity were the most abundant terms. In the cellular component ontology, cell part, membrane part, and organelle were the most abundant terms. In the biological process ontology, cellular process, biological regulation, and metabolic process were the most abundant terms (Figure 1b–f).

**Figure 2:**
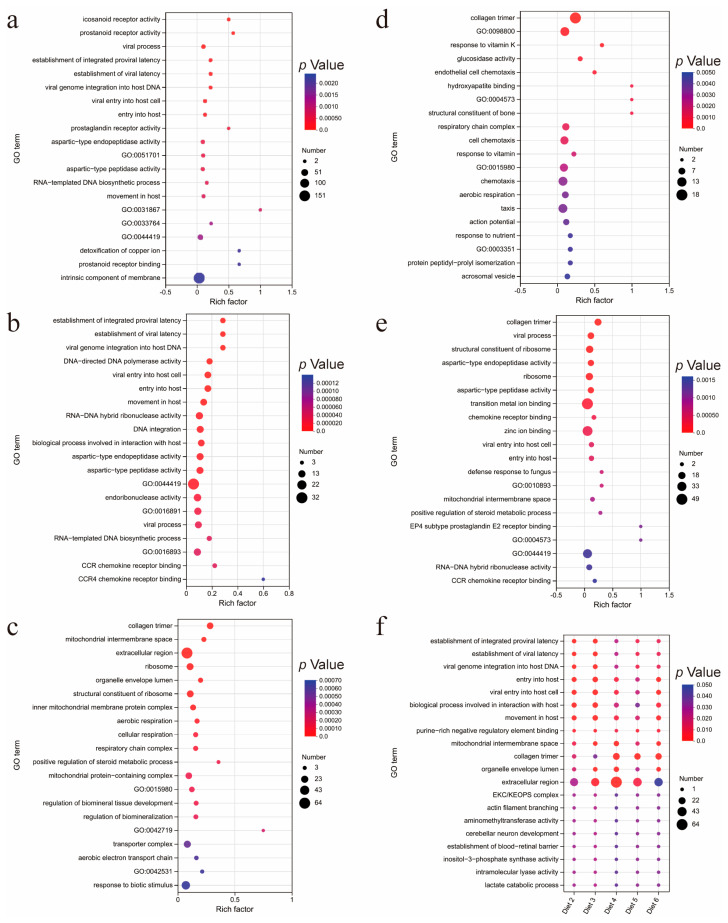
GO enrichment analysis. (**a**–**e**) The top 20 enriched items in the GO database for DEGs in the experimental and control groups, (**b**–**f**) are Diet 2, Diet 3, Diet 4, Diet 5, and Diet 6, respectively. (**f**) The overlap of the top 20 enriched GO items among the five experimental groups.

GO enrichment analysis revealed that these DEGs were enriched in 222, 344, 485, 275, and 318 GO terms, respectively, with 38 GO terms being shared among them (Supplementary Table S1). We analyzed the top 20 GO terms with the highest rich factor of DEG enrichment and found that diet 2 and diet 3 both had GO terms related to viral invasion, including viral process, establishment of viral latency, viral entry into host cell, and entry into host (Figure 2a,b). Additionally, diet 6 contained viral process, viral entry into host cell, and entry into host (Figure 2e). The top 20 GO terms in diet 4 and diet 5 did not include these four GO terms (Figure 2c,d). However, these four GO terms appeared in all five groups, which is due to the different rich factor values of the GO terms in different groups (Figure 2f).

### 3.7. KEGG Enrichment Analysis of DEGs

**Figure 3:**
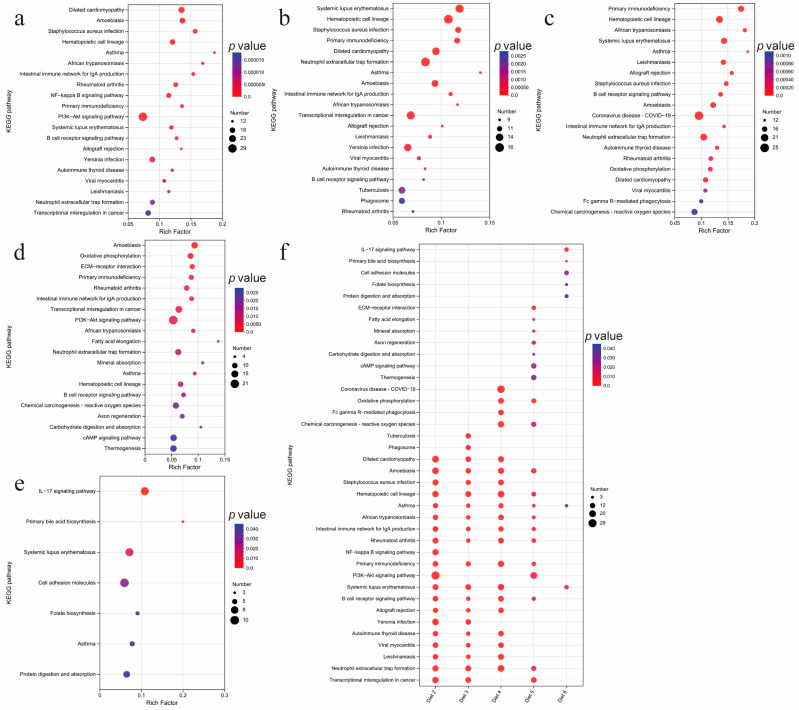
KEGG enrichment analysis. (**a**–**e**) The top 20 enriched items in the KEGG database for DEGs in the experimental and control groups, (**b**–**f**) are Diet 2, Diet 3, Diet 4, Diet 5, and Diet 6, respectively. (**f**) The overlap of the top 20 enriched KEGG pathways among the five experimental groups.

KEGG enrichment analysis revealed these DEGs showed significant enrichment in forty-two, thirty-three, forty-five, twenty-eight, and seven pathways, respectively (*p* < 0.05) (Supplementary Table S2). The top 20 KEGG pathways with the highest rich factor in each group are displayed in Figure 3a–e. Further statistical analysis of these pathways revealed that the asthma and systemic lupus erythematosus pathways were enriched in all five groups. Additionally, there were nine pathways in which DEGs from diet 2, diet 3, diet 4, and diet 5 were significantly enriched, including amoebiasis, hematopoietic cell lineage, asthma, African trypanosomiasis, intestinal immune network for IgA production, rheumatoid arthritis, primary immunodeficiency, and B cell receptor signaling pathway (Figure 3f). The main genes involved in the major pathways were *PLCB4*, *ATP6V1G3*, *LAMB3*, *SERPINB10B*, *THPO*, *ITGA5*, *ITGA2B*, *C4BPS*, *CSF3R*, *CCR10*, and *ITGB7*. Considering that there are more downregulated genes among the DEGs, the enrichment of these DEGs in these pathways may indicate that the related physiological processes of these pathways are inhibited.

### 3.8. Gene Set Enrichment Analysis

**Figure 4:**
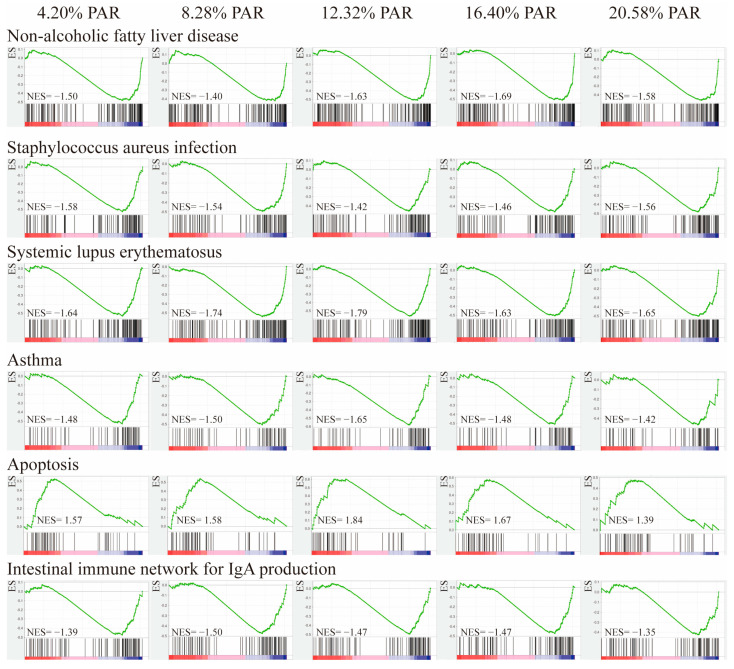
Gene set enrichment analysis. ES: Enrichment Score, reflecting the degree of enrichment of gene set members at both ends of the sorted list; NES value: Normalized Enrichment Score; The red portion corresponds to genes that are highly expressed in the experimental group, while the blue portion corresponds to genes that are highly expressed in the control group.

The GSEA enrichment analysis revealed that these DEGs were significantly enriched in 36, 33, 51, 36, and 32 pathways, respectively (*p* < 0.05), and the pathways overlapping with KEGG analysis were 19, 16, 19, 12, and 3, respectively, (Supplementary Table S3). Both the GSEA enrichment analysis and KEGG analysis revealed that the DEGs in the experimental group and the control group were significantly enriched in the pathways of systemic lupus erythematosus and asthma. GSEA revealed significant enrichment in the non-alcoholic fatty liver disease, staphylococcus aureus infection, systemic lupus erythematosus, asthma, intestinal immune network for IgA production, and apoptosis (Figure 4). Additionally, in the pathways of non-alcoholic fatty liver disease, staphylococcus aureus infection, systemic lupus erythematosus, asthma, and the intestinal immune network for IgA production, more genes were downregulated in their expression. In the apoptosis pathway, more genes were upregulated in their expression.

### 3.9. WGCNA Analysis

**Figure 5:**
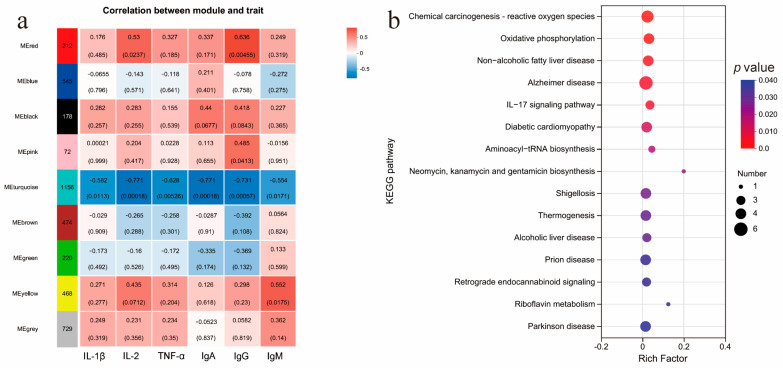
(**a**) Relationships between modules and the levels of IL-1β, IL-2, TNF-α, IgA, IgG, and IgM in the spleens of Sanhuang chickens. Each band of the matrix contains the corresponding correlation between the gene module and the levels of IL-1β, IL-2, TNF-α, IgA, IgG, and IgM in the first row and the *p* value in the second row. The intensity and direction of correlations are indicated on the right side of the heatmap (red, positively correlated; blue, negatively correlated). (**b**) KEGG enrichment results of 200 key genes.

The relationships between the levels of IL-1β, IL-2, TNF-α, IgA, IgG, and IgM in the spleen and the gene expression profile in the spleen were investigated using weighted gene coexpression network analysis (WGCNA). WGCNA revealed that all expressed genes were categorized into nine modules (Figure 5a). Specifically, genes within the MEturquoise module exhibited a negative correlation with the levels of IL-1β, IL-2, TNF-α, IgA, IgG, and IgM. Furthermore, 200 hub genes were identified within this module (Table S4). These key genes were enriched in 15 KEGG pathways, including oxidative phosphorylation, Alzheimer’s disease, non-alcoholic fatty liver disease, one carbon pool by folate, diabetic cardiomyopathy, and chemical carcinogenesis—reactive oxygen species (Figure 5b). These pathways may be involved in the regulation of immune factors in the body.

### 3.10. Differential Gene Expression in Asthma and Systemic Lupus Erythematosus Pathways

**Figure 6:**
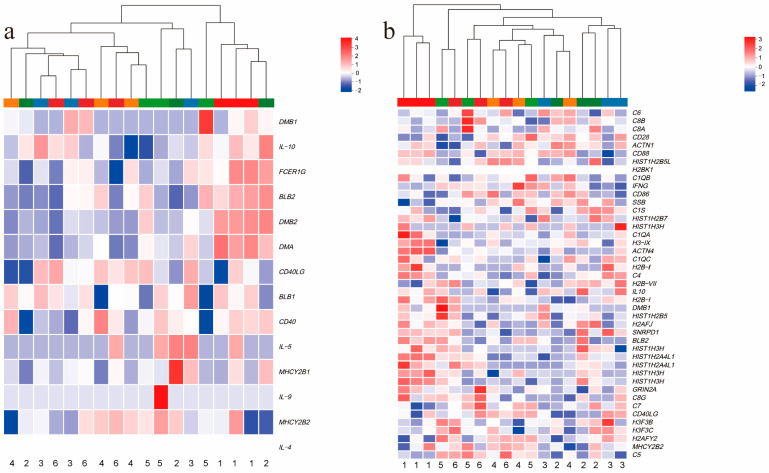
(**a**) Heatmap of important genes in the asthma pathway. (**b**) Heatmap of important genes in the systemic lupus erythematosus pathway. (**a**,**b**) The numbers below the heatmap represent the corresponding dietary groups.

Since the KEGG enrichment analysis results of DEGs from all experimental groups and control groups showed the presence of the asthma and systemic lupus erythematosus pathways, we conducted a clustering analysis of the important genes in these two pathways to understand their expression patterns under different treatments. The results of the clustering analysis indicate that the important genes in these two pathways have higher expression levels in the control group and diets 2 and 3 are overall more distant from diet 1 (Figure 6a,b). In addition, the IL-10 gene has a relatively high expression level in diet 1.

## 4. Discussion

### 4.1. PAR Can Be Used in Feed for Sanhuang Chickens

The main preparation process of PA extracts involves boiling with water, followed by the removal of impurities using ethanol of a certain volume fraction and finally refinement [40]. In this preparation process, the loss of the nutritional components in PA is very low. Previous work shows that PAR contains 56.91% crude protein, 20.48% crude fat, 15.09% crude fiber, and 3.29% crude ash [19]. This indicates that PAR has sufficient protein and can serve as a substitute for soybean meal. In this study, we detected several active peptides from PAR, including lysosome, sNPF, defensins, periplanetasin-2, periplanetasin-4, periplanetasin-5, attacins, and antimicrobial peptides. Periplanetasin-2 [10] and periplanetasin-4 [11] exhibit antimicrobial effects, defensins possess disease resistance capabilities [41], and periplanetasin-5 [12] has antitumor effects. Additionally, PA extracts are used to inhibit tumor growth [42], promote wound healing [43], and have a promoting effect on gastric ulcer rats [44]. The results suggest that using PAR as a substitute for soybean meal in feed may have a role in reducing bacterial and disease risks.

### 4.2. PAR Improved the Growth Performance of Sanhuang Chickens

Previous studies have demonstrated that PA possesses adequate nutritional components and a superior amino acid composition compared to soybean meal, with a higher ratio of essential amino acids to the total amino acid content than soybean meal [45]. Essential amino acids are key nutrients for the growth and development of animals. Feed with a high content of essential amino acids can better meet the growth needs of animals and promote their rapid growth. In addition, essential amino acids also have the benefits of improving feed efficiency, enhancing immunity, and reducing the incidence of nutritional deficiencies. In practical applications where PA has been used as an animal feed ingredient, it has been observed to significantly enhance animal growth performance [3]. Long et al. [16] show that the PAR had no negative effects on the growth performance and body composition of juvenile *Oreochromis niloticus*. However, there is a lack of relevant studies on the PAR used as a poultry feed ingredient. Diet 2 and diet 3 increased the ADG and decreased the FCR of Sanhuang chickens, while diet 4, diet 5, and diet 6 decreased the ADG and increased the FCR of Sanhuang chickens. Long et al. [16] also found that excessive PAR increased the FCR. The cause of the aforementioned results may be related to the differences in crude fiber content in various diets. An appropriate amount of crude fiber can promote gastrointestinal motility, help maintain intestinal health, and reduce the incidence of gastrointestinal diseases [46], but an excessive amount of fiber may lower the digestibility of nutrients [47]. Generally speaking, chickens have relatively short intestines and a lower content of cellulase in their digestive system; therefore, an excessive amount of cellulose in chicken feed is not conducive to the digestion and absorption of nutrients by chickens. Zuidhof et al. [48] demonstrated that diets with high fiber content can increased the FCR. An increase in the cellulose content of the diet may also lead to an increase in the content of non-starch polysaccharides. The increase in non-starch polysaccharides can raise the viscosity of the chyme in the poultry digestive tract, thereby reducing the digestibility of nutrients in the intestine [49]. The PAR contains 15.09% crude fiber, whereas the soybean meal used in the experiment has a crude fiber content of 5.20%. Every time 20% of the soybean meal in the feed is replaced with PAR, the crude fiber content in the feed increases by about 0.3%. However, the crude fiber content in the base feed is only 2.80%. The high PAR leads to a significant increase in crude fiber content in the feed, thereby increasing the FCR. Nevertheless, there is no statistically significant difference in growth performance between diet 6 and diet 1, but there are significant differences in FCR compared to diet 2 and diet 3. These results suggest that appropriate quantities of PAR should be used when incorporating it into feed, otherwise it may reduce feed utilization efficiency, leading to resource wastage.

### 4.3. PAR Increased the Levels of Immune Factors in Sanhuang Chickens

In the environment of chicken coops, feces, feathers, and air may all contain pathogens such as viruses [50,51], bacteria [52], and parasites [53]. When these pathogens are recognized by the immune system, they stimulate the secretion of various cytokines, including IL-1, IL-6, and TNF-α, thereby inducing immune and oxidative stress [54,55]. The increase in the levels of Il-1, IL-2, and TNF-α in the body represents an enhancement of individual immunity [56]. In this study, the concentrations of IL-1β, IL-2, and TNF-α in the spleen and serum of the experimental groups were greater than those in the control group. This indicates that the PAR has enhanced the immunity of the Sanhuang chickens. Additionally, previous studies have reported that PA extracts can increase the concentrations of IL-1β and TNF-α [57] and increase the level of IL-2 [58]. The research by Long et al. [16] also indicates that PAR significantly enhances the immunity of O. niloticus fry. Our research is consistent with the results of these studies.

Additionally, with respect to the high-PAR diet, the concentrations of IL-1β, IL-2, and TNF-α were negatively correlated with the dietary PAR content, suggesting that high doses of PAR result in a decreased positive effect on Sanhuang chicken. The mechanism behind the decreased positive effect is currently unclear. However, other studies have also shown that extracts of PA can inhibit the production of IL-1β, IL-6, and TNF-α [59,60].

Furthermore, the PAR also contains abundant chitin. Previous studies have shown that chitosan can increase the levels of IL-1, IL-6, and TNF-α, but excessive chitosan can diminish these effects [61,62]. PAR contains approximately 13% polysaccharides, which may explain the increase in PAR content in the feed and the decrease in the levels of IL-1, IL-2, and TNF-α. Notably, chitosan has similar regulatory effects on immunoglobulins [63,64]. Similar explanations will not be provided in the following sections.

Immunoglobulins are the principal operators of the adaptive humoral immune response [65]. Serum immunoglobulins mainly include IgM, IgA, and IgG, which are important participants in humoral immunity. An increase in their levels indicates an enhancement of the body’s immune function. Long et al. [5] showed that appropriate substitution of PA for fish meal as a dietary protein can increase the content of IgM in rainbow trout serum. She et al. [3] found that adding PA powder to feed for chickens can increase the levels of IgA, IgG, and IgM in broiler chickens. However, excessive addition can reduce this positive effect. Our results are consistent with previous studies: PAR increased the concentrations of IgA, IgG, and IgM in the spleen and serum, and this positive effect diminished as the PAR level increased. The results indicate that substances in the PAR may participate in the humoral regulation of Sanhuang chickens.

### 4.4. PAR Enhanced the Antioxidant Capacity of Sanhuang Chickens

Oxidative stress refers to the condition in which the body experiences excess production of reactive oxygen species (ROS) due to harmful stimuli, causing the oxidation level in the body to exceed the clearance capacity of antioxidants, thereby leading to an imbalance in the body’s antioxidant system [66]. The antioxidant defense system, which includes the enzymes T-AOC, SOD, CAT, and GSH-Px, is crucial for counteracting oxidative damage. These antioxidant enzymes are considered sensitive indicators of oxidative stress in chickens [67]. Additionally, the content of MDA could be used as a biomarker for evaluating the degree of lipid peroxidation [68]. Previous studies have shown that dietary supplementation with PA meal or PA powder could improve the antioxidant capacity and immunity of fish [5] and broilers [3]. The PA extract showed antioxidant activity in a dose-dependent manner [60].

The experimental results of this study indicated that replacing soybean meal with PAR significantly increased the levels of GSH-Px, T-AOC, and CAT in both the serum and liver. Excessive PAR in feed reduces its positive impact on antioxidant capacity. This is consistent with previous research results. Long et al. [16] also reported that excessive PAR reduces antioxidant capacity. These results indicate that replacing soybean meal in feed with PAR has a positive effect on enhancing the antioxidant capacity of Sanhuang chickens. The mechanism of this effect may be related to the peptide periplanetasin-2. In this study, periplanetasin-2 was detected in the PAR. Yun et al. [10] demonstrated that planetasin-2, which possesses strong antimicrobial activity, induces intracellular ROS production, leading to oxidative damage within cells. This could also explain the increase in MDA in the experimental group as the content of PAR increases. These results indicate that the effect of PAR on antioxidant capacity is dose dependent, with the optimal enhancement observed with diet 2.

### 4.5. Transcriptomic Analysis Revealed That PAR Downregulates Multiple Disease-Related Pathways

Transcriptome sequencing can be used to detect the gene expression levels of a species under any condition, screen and analyze DEGs, explore relevant physiological pathways, and understand their associated mechanisms [69]. The environment of poultry farms typically harbors a large number of viruses, bacteria, fungi, or parasites. The presence of these pathogens may trigger various diseases. Viruses and bacteria can also induce respiratory diseases in chickens. Infection by these pathogens may trigger immune system responses in chickens, leading to immune system dysregulation or overactivation, thereby causing immune-related diseases [70]. The spleen, as a vital organ of the immune system, plays a crucial role in regulating immune responses, maintaining homeostasis, and resisting diseases. Analyzing the transcriptome of the spleen in Sanhuang chickens enables a more comprehensive understanding of the impact of American cockroach residue on immune function and physiological status [71,72], thereby providing important clues for further exploration of the underlying mechanisms involved. In this study, we found that there were differences in the expression levels of spleen genes between the experimental group and the control group of Sanhuang chickens and that more of these GEGs were downregulated. This suggests that PAR may inhibit the expression of certain genes in Sanhuang chickens, thereby affecting the immune and antioxidant capabilities of the chickens. The spleen is not the main organ for regulating antioxidant function, so there are not many DEGs related to antioxidant function. No pathways related to antioxidants were identified in subsequent analyses. Despite this, we have found that heat shock protein (*HSPB9*) and *PLCB4* was uniformly downregulated in the experimental group. In response to cellular stress, such as heat and oxidative stress, the expression of HSPs is upregulated to maintain cellular homeostasis [73]. *PLCB4* is also considered to be related to heat stress [74]. This can also serve as an explanation for the enhancement of antioxidant capacity.

The GO annotation analysis revealed that the DEGs in the experimental group were mainly annotated in the GO terms of binding, catalytic activity, cell part, membrane part, and organelle, cellular process, biological regulation, and metabolic process. This suggests that the DEGs affected by PAR may be related to the process of substance import and export in cells, biological regulation, and cellular metabolism. Additionally, the GO enrichment analysis results show that the DEGs in both the experimental and control groups are enriched in viral invasion pathways, including viral process, establishment of viral latency, viral entry into host cell, and entry into host. Viruses are intracellular parasites, which means that at the beginning of each new infection cycle, the virus must have a way to transport its genetic material and other required components through the host cell barrier to the replication site [75]. This can also serve as an explanation for why DEGs are annotated to a large number of terms related to the cell membrane.

KEGG enrichment analysis revealed that DEGs in both the experimental and control groups were enriched in pathways related to immune disease, immune system, and infectious disease, including bacteria and parasites, including amoebiasis, hematopoietic cell lineage, asthma, African trypanosomiasis, intestinal immune network for IgA production, rheumatoid arthritis, primary immunodeficiency, and B cell receptor signaling pathway. Since, in this study, more DEGs are downregulated, we speculate that these pathways are inhibited by PAR. We used GSEA to further verify our hypothesis. The GSEA results indicate that PAR downregulates the expression of genes associated with disease-related pathways and upregulates the expression of genes associated with apoptosis pathways. The results of the GSEA confirmed our hypothesis. These results suggest that the active substances in PAR may enhance the resistance of Sanhuang chickens to viruses, bacteria, and parasites. The main genes involved in the major pathways were *PLCB4*, *ATP6V1G3*, *LAMB3*, *SERPINB10B*, *THPO*, *ITGA5*, *ITGA2B*, *C4BPS*, *CSF3R*, *CCR10*, and *ITGB7*.

*ATP6V1G3* are probably related to bone density [76]. Laminin subunit beta-3 (*LAMB3*) plays a crucial role in the development of chicken skeletal muscle and cell differentiation [77]. *PLCB4* is believed to be related to the regulation of calcium and phosphorus absorption and metabolism [78]. The upregulation of *PLCB4* expression may affect the activation of calcium/calmodulin-dependent protein kinase (CAMKs) through changes in Ca^2+^ concentration [79]. These results suggest that PAR may affect the growth performance of Sanhuang chicken by regulating the expression levels of these genes, thereby influencing the nutrient absorption and growth of Sanhuang chicken. In addition, *PLCB4* is also considered to be related to immune response [74]. Polansky et al. [80] found that the content of *SERPINB10* protein increased in chicken liver and serum invaded by Salmonella. Human thrombopoietin (*THPO*) stimulates the proliferation and maturation of megakaryocytes and controls the production of platelets [81]. Platelets participate in important physiological functions such as immunity and hematopoietic regulation. *ITGA5* is abnormally expressed in various tumors and is closely related to the adhesion, migration, and invasion of tumor cells, as well as the generation of tumor blood vessels [82]. *ITGA2B* is expressed in platelets and participates in platelet aggregation, which is crucial for maintaining blood fluidity and preventing bleeding [83]. The protein encoded by *ITGB7* plays an important role in the migration of lymphocytes to the intestinal mucosa and lymph nodes and binds to ligands such as E-cadherin. *C4BP*, as a complement inhibitor, can promote apoptosis, but when C4BP functions in a complement-independent manner, it can promote cell survival, prevent autoimmune damage, and regulate the virulence of microbial pathogens [84]. The protein encoded by the *CSF3R* gene is the receptor for colony-stimulating factor 3 (G-CSF), which is a cytokine that controls the production, differentiation, and function of granulocytes, such as neutrophils. *CCR10* plays a crucial role in regulating the localization and function of immune cells within tissues, participating in the coordination of humoral and cellular immunity [85]. These results indicate that PAR may regulate the immune function of Sanhuang chickens through these genes.

All the differential genes in the experimental groups and the control group were enriched in the asthma and systemic lupus erythematosus pathways. When analyzing the key genes in these two pathways, it was found that the IL-10 gene had a higher expression level in the control group. IL-10 can inhibit the production of inflammatory mediators, thereby reducing the severity of the inflammatory response [86], while also possessing tumor-promoting effects [87]. The control group expressing more IL-1 can suggest that it faced more inflammatory challenges. Both IL-10 and IL-1 have pro-inflammatory effects in systemic lupus erythematosus and during inflammation or autoimmune diseases, and IL-10 inhibits the production of IL-1 by monocytes, macrophages, etc. [88], which provides evidence for the lower level of IL-1 in the control group compared to the experimental group. In addition, IL-10-producing B cells have diverse states that are induced from multiple B cell subsets [89]. The results of the KEGG enrichment analysis show that the DEGs of all experimental groups and the control group were enriched in the B cell receptor signaling pathway. This may indicate that PAR regulates IL-10 by controlling the B cell receptor signaling pathway, thereby affecting the levels of IL-10 in the serum and spleen. GSEA enrichment analysis reveals that genes related to the apoptosis pathway are more expressed in the experimental group. TNF-α plays a crucial role in the body’s fight against tumors by inducing apoptosis [90]. Infections with viruses, bacteria, and parasites, as well as weakened immune system function, can all lead to the occurrence of tumors. The peptides periplanetasin-2 [10] and periplanetasin-4 [11] detected in PAR possess antibacterial properties, defensins have antipathogenic capabilities [41], and periplanetasin-5 [12] exhibits antitumor effects. These studies provide evidence for the antitumor capabilities of PAR. They also explain the reason for the elevated TNF-α levels in the experimental group.

Although in this study, immune factors, antioxidant indicators, and transcriptome analysis all demonstrated that PAR has a positive effect on the immune and antioxidant capabilities of Sanhuang chickens, we also observed the same situation as previous studies: excessively high levels of PAR can reduce these positive effects and also impact growth performance, increasing the FCR [5,16,19]. Such impacts are not conducive to the full utilization of PAR; therefore, based on previous research [5,16,19], we suggest that the PAR content should not exceed 60% during its application. In subsequent research, on the one hand, it is necessary to continue to reduce the differences in PAR among different feeds to achieve more efficient utilization of PAR. On the other hand, the research target can be shifted to laying hens. Adding a certain level of fiber to the diet can have a positive impact on the growth performance and egg quality of laying hens. PAR has a higher crude fiber content than soybean meal, which may produce even better effects when applied to laying hens.

## 5. Conclusions

This study found that an appropriate substitution of PAR for soybean meal improved the immune function and antioxidant capacity of Sanhuang chickens without affecting their growth performance, and these levels were optimal at diet 2. Transcriptomic analysis revealed that replacing soybean meal with PAR reduced the expression of genes associated with disease-related pathways. This suggests that using an appropriate amount of PAR to replace soybean meal is feasible. It provides a cost-effective new method for the application of insect protein.

## Figures and Tables

**Table 1:** The ingredients and nutritional composition of the experimental diets (%).

Ingredient	Diets					
	Diet 1	Diet 2	Diet 3	Diet 4	Diet 5	Diet 6
Corn	63.15	64.83	66.81	69.72	71.23	72.80
Wheat bran	1.80	2.00	2.00	2.00	2.80	3.53
Soybean meal	23.50	18.80	14.10	9.40	4.70	0.00
PAR	0.00	3.32	6.64	9.83	13.02	16.22
Calcium hydrogen phosphate	1.60	1.40	1.20	1.00	1.00	1.00
DL-Methionine	0.60	0.60	0.60	0.60	0.60	0.60
Vegetable oil	3.50	3.00	2.40	1.50	1.00	0.50
Premix ^1^	5.00	5.00	5.00	5.00	5.00	5.00
Salt	0.25	0.25	0.25	0.25	0.25	0.25
Choline chloride	0.20	0.20	0.20	0.20	0.20	0.20
Talcum powder	0.50	0.50	0.50	0.30	0.40	0.30
Total	100.00	100.00	100.00	100.00	100.00	100.00
Nutrient composition ^2^						
Metabolizable energy ^3^, MJ/kg	12.13	12.13	12.13	12.13	12.13	12.13
Crude protein	16.01	16.01	16.01	16.01	16.01	16.01
Calcium	0.70	0.70	0.70	0.70	0.70	0.70
Phosphorus	0.60	0.60	0.61	0.61	0.62	0.62
Nonphytate phosphorus	0.43	0.44	0.44	0.44	0.45	0.45
Methionine	0.84	0.84	0.84	0.84	0.84	0.84
Lysine	0.77	0.77	0.78	0.78	0.79	0.79
Crude fiber	2.80	3.09	3.40	3.72	4.03	4.35

^1^ The premix provided the following per kg of diet: VA 8000 IU, VD3 3000 IU, VE 16 IU, VK3 2 mg, VB1 1.6 mg, VB2 8.3 mg, VB6 2.6 mg, VB12 0.015 mg, nicotinic acid 43 mg, calcium pantothenate 12 mg, folic acid 0.92 mg, biotin 0.12 mg, choline 530 mg, Cu 8 mg, Fe 80 mg, Mn 60 mg, Zn 80 mg, I 0.35 mg, and Se 0.15 mg. ^2^ The metabolizable energy is calculated and the others are measured values. ^3^ The metabolic energy was calculated according to the NRC [30].

**Table 2:** The detection results of the polypeptide content of PAR.

Items	Test Results
Total polysaccharides, %	13.93
Lysosom, µg/L	13.43
sNPF, ng/L	85.22
Defensins, ng/L	1338.53
Periplanetasin-2, pg/mL	135.93
Periplanetasin-4, pg/mL	87.21
Periplanetasin-5, pg/mL	52.97
Attacins, pg/mL	34.67
Antimicrobial peptides, pg/mL	201.27

**Table 3:** Growth performance of Sanhuang chickens.

Items	ADG (g)	95% Confidence	ADFI (g)	95% Confidence	FCR	95% Confidence
Diet1	34.99 ± 4.57 ^ab^	[29.32, 40.67]	124.51 ± 13.97	[107.17, 141.86]	3.59 ± 0.13 ^ab^	[3.43, 3.75]
Diet2	34.88 ± 1.32 ^ab^	[33.23, 36.52]	119.95 ± 6.06	[112.42, 127.48]	3.50 ± 0.14 ^b^	[3.32, 3.67]
Diet3	36.56 ± 3.16 ^a^	[32.64, 40.49]	127.34 ± 6.26	[119.56, 135.11]	3.54 ± 0.26 ^b^	[3.21, 3.87]
Diet4	33.02 ± 2.52 ^ab^	[29.89, 36.16]	122.79 ± 9.62	[110.84, 134.74]	3.76 ± 0.35 ^ab^	[3.33, 4.20]
Diet5	32.56 ± 3.85 ^ab^	[27.78, 37.35]	119.35 ± 10.22	[106.66, 132.03]	3.71 ± 0.36 ^ab^	[3.26, 4.16]
Diet6	30.34 ± 2.94 ^b^	[26.69, 34.00]	119.90 ± 4.31	[114.55, 125.26]	3.98 ± 0.29 ^a^	[3.62, 4.33]
SEM	2.04		5.7		0.17	
ANOVA	0.074		0.684		0.10	
Linear	0.009		0.376		0.01	
Quadratic	0.012		0.604		0.017	

^a,b^ means with no common superscripts differ significantly (*p* < 0.05). Abbreviations: ADG, average daily gain; ADFI, average daily feed intake; FCR, feed conversion ratio; SEM, standard error of the mean. The data are presented as mean ± standard deviation.

**Table 4:** Immune parameters of spleens and serum.

Items	Diet						SEM	*p* Value			Eta Squared
	1	2	3	4	5	6		ANOVA	Linear	Quadratic	
Spleen											
IL-1β, ng/L	118.71 ^c^	144.99 ^a^	127.30 ^bc^	130.22 ^b^	131.77 ^b^	127.30 ^bc^	2.155	0.001	0.893	0.298	0.780
IL-2, ng/L	171.95 ^d^	206.59 ^a^	195.85 ^b^	199.40 ^ab^	183.26 ^c^	193.67 ^b^	2.909	<0.001	0.496	0.062	0.884
TNF-α, ng/L	72.57 ^c^	93.21 ^a^	81.27 ^b^	81.14 ^b^	81.42 ^b^	80.11 ^b^	1.591	<0.001	0.949	0.244	0.848
IgA, μg/mL	7.96 ^c^	9.43 ^a^	8.60 ^b^	9.16 ^ab^	8.87 ^ab^	8.87 ^ab^	0.132	0.004	0.213	0.089	0.728
IgG, μg/mL	77.39 ^c^	88.32 ^a^	86.87 ^ab^	89.60 ^a^	84.56 ^ab^	81.41 ^bc^	1.208	0.005	0.654	<0.001	0.713
IgM, μg/mL	5.18 ^c^	6.92 ^a^	5.97 ^b^	5.79 ^b^	5.94 ^b^	6.01 ^b^	0.137	<0.001	0.741	0.440	0.813
Serum											
IL-1, ng/L	129.79 ^c^	152.20 ^a^	133.29 ^bc^	141.02 ^b^	128.63 ^c^	138.62 ^bc^	2.223	0.002	0.692	0.732	0.765
IL-2, ng/L	190.49 ^b^	224.29 ^a^	196.60 ^b^	199.58 ^b^	194.70 ^b^	192.07 ^b^	3.296	0.006	0.261	0.254	0.706
TNF-α, ng/L	77.90 ^c^	98.88 ^a^	87.51 ^b^	82.35 ^bc^	85.98 ^b^	85.67 ^b^	1.670	<0.001	0.889	0.488	0.866
IgA, μg/mL	7.99 ^c^	10.52 ^a^	9.37 ^b^	9.50 ^b^	9.26 ^b^	9.50 ^b^	0.188	<0.001	0.321	0.102	0.909
IgG, μg/mL	82.15 ^c^	96.69 ^a^	87.63 ^bc^	87.64 ^bc^	91.68 ^ab^	86.42 ^bc^	1.311	0.007	0.820	0.293	0.702
IgM, μg/mL	5.60 ^d^	7.79 ^a^	6.40 ^bc^	6.66 ^b^	6.55 ^bc^	6.26 ^c^	0.162	<0.001	0.968	0.136	0.947

^a–d^ Within a row, means with no common superscripts differ significantly (*p* < 0.05). Abbreviations: IL-1β, interleukin 1 beta; IL-2, interleukin 2; TNF-a, tumor necrosis factor alpha; IgA, immunoglobulin A; IgG, immunoglobulin G; IgM, immunoglobulin M.

**Table 5:** Antioxidant capacity of liver and serum.

Items	Diet						SEM	*p*-Value			Eta Squared
	1	2	3	4	5	6		ANOVA	Linear	Quadratic	
Liver											
GSH-Px (ng/L)	46.28 ^b^	56.62 ^a^	55.09 ^a^	53.85 ^a^	55.04 ^a^	50.88 ^ab^	1.093	0.036	0.464	0.021	0.590
T-AOC (U/mL)	6.86 ^c^	8.58 ^a^	7.60 ^b^	8.08 ^ab^	7.60 ^b^	7.72 ^b^	0.140	<0.001	0.542	0.129	0.819
MDA (nmol/L)	14.67 ^a^	10.74 ^c^	12.96 ^b^	13.04 ^b^	12.45 ^b^	12.61 ^b^	0.294	<0.001	0.411	0.279	0.904
SOD (U/mL)	84.29	92.67	89.36	89.11	92.13	89.82	1.076	0.278	0.254	0.270	0.376
CAT (U/mL)	47.98 ^c^	57.59 ^a^	49.35 ^bc^	49.14 ^bc^	50.40 ^bc^	53.61 ^ab^	0.939	0.004	0.750	0.919	0.726
Serum											
GSH-Px (ng/L)	50.25 ^c^	66.44 ^a^	56.36 ^b^	58.90 ^b^	59.16 ^b^	56.94 ^b^	1.405	0.010	0.637	0.256	0.681
T-AOC (U/mL)	6.49 ^c^	8.31 ^a^	7.24 ^b^	7.49 ^b^	7.62 ^b^	7.20 ^b^	0.141	<0.001	0.564	0.109	0.867
MDA (nmol/L)	13.63 ^a^	8.75 ^c^	10.50 ^b^	10.38 ^b^	10.29 ^b^	10.07 ^bc^	0.387	<0.001	0.094	0.034	0.854
SOD (U/mL)	83.52 ^c^	106.06 ^a^	98.00 ^b^	99.16 ^b^	97.90 ^ab^	94.24 ^b^	1.830	<0.001	0.436	0.018	0.809
CAT (U/mL)	51.20 ^c^	66.00 ^a^	58.42 ^b^	60.98 ^b^	60.86 ^b^	59.80 ^b^	1.167	<0.001	0.217	0.067	0.839

^a–c^ Within a row, means with no common superscripts differ significantly (*p* < 0.05). Abbreviations: GSH, glutathione peroxidase; MDA, malondialdehyde; SOD, superoxide dismutase; CAT, catalase; T-AOC, total antioxidant capacity.

## Data Availability

The datasets analyzed during the current study are available from the corresponding author upon reasonable request. The raw data of the transcriptomes were uploaded to the NCBI SRA database (sequence number: PRJNA1080808).

## Supplementary Materials

The following supporting information can be downloaded at: https://www.mdpi.com/article/10.3390/ani15010094/s1, Table S1. The GO enrichment analysis results for DEGs. Table S2. The KEGG enrichment analysis results for DEGs. Table S3. GSEA Analysis Results. Table S4. 200 key genes negatively correlated with immune factors.

## Abbreviations

PAR	*Periplaneta americana* residue
PA	*Periplaneta americana*
IL-1β	Interleukin 1 beta
IL-2	Interleukin 2
TNF-α	Tumor necrosis factor-alpha
IgA	Immunoglobulin A
IgM	Immunoglobulin M
IgG	Immunoglobulin G
cPLA_2_	Cytosolic phospholipase A_2_
AA	Arachidonic acid
COX	Cyclooxygenase
LOX	Lipoxygenase
LTB_4_	Leukotriene B_4_
PGE_2_	Prostaglandin E_2_
MDA	Malondialdehyde
T-AOC	Total antioxidant capacity
CAT	Catalase
SOD	Superoxide dismutase
GSH-Px	Glutathione peroxidase
GO	Gene Ontology
KEGG	Kyoto Encyclopedia of Genes and Genomes
GSEA	Gene set enrichment analysis
WGCNA	Weighted gene coexpression network analysis
